# The Predictability of Preoperative Pilocarpine-Induced Lens Shift on the Outcomes of Accommodating Intraocular Lenses Implanted in Senile Cataract Patients

**DOI:** 10.1155/2016/6127130

**Published:** 2016-07-19

**Authors:** Jin Li, Qi Chen, Zhibo Lin, Lin Leng, Fang Huang, Ding Chen

**Affiliations:** Eye Hospital, Wenzhou Medical University, Wenzhou, Zhejiang 325027, China

## Abstract

*Purpose*. To evaluate the predictability of lens shift induced by pilocarpine (LS_Pilo_) on the outcomes of accommodating intraocular lens (Acc-IOL) implantation.* Methods*. Twenty-four eyes of 24 senile cataract patients who underwent phacoemulsification and Acc-IOL implantation were enrolled. LS_Pilo_ was evaluated with anterior segment optical coherence tomography (AS-OCT). At 3 months postoperatively, the best corrected distance visual acuities (BCDVA), distance-corrected near visual acuities (DCNVA), and subjective and objective accommodations were measured. IOL shifts under accommodation stimulus (IOLS_Acc_) were evaluated with AS-OCT.* Results*. The mean LS_Pilo_ was 112.29 ± 30.72 *µ*m. LS_Pilo_ was not associated with any preoperative parameters. The mean IOLS_Acc_ was 130.46 ± 42.71 *µ*m. The mean subjective and objective accommodation were 1.54 ± 0.39 D and 1.27 ± 0.41 D, respectively. The mean postoperative BCDVA and DCNVA (log MAR value) were 0.22 ± 0.11 and 0.24 ± 0.12, respectively. LS_Pilo_ positively correlated with IOLS_Acc_ (*r* = 0.541; *P* = 0.006), subjective accommodation (*r* = 0.412; *P* = 0.022), and objective accommodation (*r* = 0.466; *P* = 0.045), respectively.* Conclusion*. LS_Pilo_ is an independent preoperative parameter associated with the postoperative Acc-IOL mobility and pseudophakic accommodation. It may offer valuable information for ophthalmologists in determining the suitable candidates for Acc-IOL implantation.

## 1. Introduction

Restoration of accommodation in pseudophakic patients is a major goal of modern cataract surgery. So far a variety of devices and surgical techniques have been introduced to solve the problem of presbyopia correction and to provide satisfactory distance and near vision without spectacles after cataract surgery. An example is implantation of multifocal intraocular lenses (MF-IOLs) with diffractive or refractive optic designs that improve uncorrected near vision at the expense of reduced contrast sensitivity, halos around lights, and night glare [1–3]. To overcome these visual side effects inducted by MF-IOLs, the so-called accommodating intraocular lenses (Acc-IOLs) were developed [4].

The mechanism of the current available Acc-IOLs is based on the Helmholtz theory of accommodation [5], which assumes that the force is transmitted from the ciliary muscle to the lens via the zonular apparatus. These IOLs are designed to transform such forces of the ciliary muscle into a forward shift of the IOL optic through the incorporates hinges, therefore increasing the effective refraction power for near vision [6]. However, the outcomes vary dramatically among patients implanted with Acc-IOLs [6–10]. Most patients achieved both satisfactory distance and near vision without spectacles, while some patients gained very limited accommodation and near vision. Multiple factors may contribute to the variation in the outcomes of Acc-IOLs among individuals [11–14]. On the basis of the optic-shift concept, the postoperative accommodation of an Acc-IOL is dependent on ciliary muscle contraction. We hypothesize that the individual differences in the function of ciliary muscle may play a key role in the variation of Acc-IOLs implanting outcomes.

Unfortunately, there is no established method to assess the potential of ciliary muscle contraction before cataract surgery. It is well known that there is an increase of curvature and anterior movement of the crystalline lens during accommodation [15]. In senile cataract patients, the lens loses its deformability, while the axial movement of cataractous lens driven by ciliary muscle contraction may still exist [16, 17], which may work as a function of contractile ability of the ciliary muscle. So we hypothesize that the preoperative evaluation of the lens mobility may indicate the potential contraction power of ciliary muscle related to the postoperative accommodation of an Acc-IOL. Pilocarpine induced lens shifts have been used to assess accommodation in phakia and pseudophakia in previous studies [8, 18], and it might be applicable to senile cataract patients.

The aim of this study was to evaluate the preoperative pilocarpine-induced lens shift, and its predictability on the outcomes of Acc-IOL implantation in senile cataract patients. To the best of our knowledge, for the first time, the accommodation potential assessment is incorporated into the preoperative evaluations of cataract surgery and is considered as a possible indication for Acc-IOL implantation.

## 2. Patients and Methods

This study is a prospective case series. Eligible patients were enrolled at Cataract Department of the Eye Hospital of Wenzhou Medical University from December 2013 to November 2014. Inclusion criteria were age-related cataract and good overall physical constitution. Exclusion criteria were corneal astigmatism of 1.50 diopters (D) or more, a history of ocular trauma or intraocular surgery, laser treatment, diabetes mellitus requiring medical control, pseudoexfoliation syndrome, glaucoma, uveitis, and retinal pathology that would limit postoperative restoration of vision. Patients who did not return for prescribed follow-ups or had any intraoperative complications were removed from the study.

The study was approved by the Ethics Committee and the Institutional Review Boards of Wenzhou Medical University. All researches and measurements followed the tenets of the Declaration of Helsinki, and informed consents were obtained from all patients.

### 2.1. Preoperative Evaluation

Each patient underwent a complete eye examination including visual acuity, manifest refraction, keratometry, slit lamp microscope, intraocular pressure (IOP), and dilated retinal examination. Pupil diameter was measured using a template rule (Matheson Optometrists, UK) under standard room light before any eye drop was administered. The LOCS III [19] nuclear opacity was graded on a scale of 0.1 to 6.9 by comparing a digital photograph of each lens with standard color photographic transparencies of nuclear opalescence (NO) and nuclear color (NC) using a slit lamp after dilation by the same ophthalmologist. Biometry including corneal curvature, corneal diameter (white-to-white, WTW), and axial length were obtained with the IOLMaster device (Carl Zeiss Meditec AG, Jena, Germany). The required IOL power was calculated using the SRK/T formula with a postoperative refractive target between plano and −0.50 D.

The central corneal thickness (CCT) and lens shift induced by pilocarpine (LS_Pilo_) were evaluated with an anterior segment optical coherence tomography (AS-OCT, Visante-1000, Carl Zeiss Meditec., Dublin, Germany). The device was calibrated with an artificial eye before each measurement. The anterior chamber depths (ACDs, distance from the anterior surface of the lens to the corneal vertex) both in the baseline unaccommodated state and after induced accommodation with 2% pilocarpine drops (one drop placed every 5 minutes, 6 times in total prior to testing) were measured. The washout time between application of pilocarpine and dilation eye drops should be at least 12 hours. Examination of the intraocular distances was made with the device's software by manually set calipers. LS_Pilo_ was defined as the Δ-values of these two ACDs, LS_Pilo_ = ACD_Baseline_ − ACD_Pilo_. The measurement was repeated 3 times.

### 2.2. Surgical Procedure

All surgeries were performed by a single surgeon (DC) under topical anesthesia. A 2.75 mm watertight clear corneal incision was made and 5.0 to 6.0 mm continuous curvilinear capsulorhexis was performed. The nucleus was removed by phacoemulsification in the capsular bag and the residual cortex was aspirated with the I/A handpiece. Viscoelasticity was then used to inflate the capsular bag, and a piece of monooptic Acc-IOL TetraFlexHD® (Lenstec Inc., St. Petersburg, FL, USA) was slowly injected into the bag. The lens was then moved back and forth along the long axis of the lens with a lens hook to verify that the leading and trailing haptics were properly positioned in the capsular bag. Viscoelasticity was then removed and the wound was verified for stability. All patients received the same postoperative regimen of antibiotic and corticoid eye drops (tobramycin + dexamethasone) (Tobradex® Ophthalmic Suspension, Alcon Laboratories Inc., Fort Worth, Texas) for 4 weeks at a dose of 4 times daily initially and then in tapered doses.

### 2.3. Postoperative Evaluation

At 3 months after surgery, each eye underwent postoperative examinations including visual acuities, manifest refraction, slit lamp, IOP, pupil diameter, CCT, corneal curvature, corneal diameter (WTW), and axial length. Visual acuities were measured using Early Treatment Diabetic Retinopathy Study (ETDRS) charts for distance and near vision at 6 m and 40 cm. Uncorrected distance visual acuities (UCDVA), best corrected distance visual acuities (BCDVA), uncorrected near visual acuities (UCNVA), and distance-corrected near visual acuities (DCNVA) were documented in logarithm of the minimum angle of resolution (log MAR) units.

The amplitudes of subjective and objective accommodation under the maximum near stimulus were measured. Subjective near-point accommodation (NPA) was measured with the defocus method. This method involved placing a lens in front of the eye that created a +3.00 D add over the best distance refraction. The reading target was placed 0.33 m away from the eye. The patient was asked to focus on the line just above the lowest discernable line, and −0.25 D spherical lenses were sequentially placed in front of the eye until the visual target blurred. The NPA power was the sum of the diopters of all minus lenses added until the target blurred. The objective accommodation was measured with the Optical Quality Analysis System (OQAS®, Visiometrics, Terrassa, Spain). OQAS is the instrument used for objective measurement of optical quality and intraocular scattering as well as objective accommodation in a clinical setting [20, 21]. Its good repeatability and reproducibility have been proven [22]. With full correction of distance visual acuity, subjects were seated at the instrument with their head stabilized in the chin rest and forehead strap. After dimming the lights in the room, subjects viewed the 20/40 sized near target at 33 cm. Defocus stimuli from +1 to −5 D in 0.5-D increments were presented to the patients; the subject was requested to fixate the target and keep it as clear as possible while the objective refraction measurement was made. The measurement was repeated 3 times. Patients were requested to shut their eyes after each measurement with the intent to relax after accommodation.

Measurement of the IOL shift under accommodation stimulus (IOLS_Acc_) was performed similarly to the method as described for measuring preoperative LS_Pilo_ using AS-OCT (Visante-1000, Carl Zeiss Meditec., Dublin, Germany). Baseline ACD measurements were performed with the patient fixating on an internal target collimated to infinity with the eye being measured. Positive and negative lenses ranging from +1 to −5 D in 0.5-D increments were presented within the OCT device to defocus the fixation target; this way, a stimulus for physiologic accommodation was provided. Patients were requested to fixate on the internal target until they were able to see as clear as possible. Then ACD measurements were performed. IOLS_Acc_ was defined as the Δ-values of these two ACDs, IOLS_Acc_ = ACD_Baseline_ − ACD_Acc_. The measurement was repeated 3 times.

### 2.4. Statistical Analysis

Statistical analyses were performed using statistical software (SPSS for Windows, V.16.0 SPSS Science, Chicago, Illinois, USA). The data is presented as the mean value ± SD. Student's *t*-test was used to assess the change of the parameters. Pearson correlation analysis was used to assess the correlations between the parameters. Multivariate linear regression analyses were performed to determine the associated preoperative factors for LS_Pilo_, postoperative accommodation, and DCNVA. A *P* value of 0.05 or less was considered statistically significant.

## 3. Results

**Figure 1:**
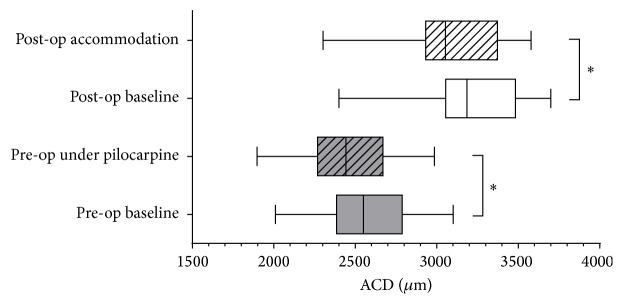
Preoperative lens shifts induced by pilocarpine (LS_Pilo_) and postoperative intraocular lens shifts under an accommodation stimulus (IOLS_Acc_) measured with anterior segment optical coherence tomography (AS-OCT). The shift is defined as the Δ-values of the anterior chamber depths (ACDs): LS_Pilo_ = ACD_Baseline_ − ACD_Pilo_, and IOLS_Acc_ = ACD_Baseline_ − ACD_Acc_. Pilo: pilocarpine; Acc: accommodation. ^*∗*^ *P* < 0.05.

This study enrolled 24 eyes of 24 patients (14 women and 10 men).  Table 1 shows the patients' preoperative characteristics. The mean preoperative baseline ACD was 2565.60 ± 305.50 *μ*m, and the mean ACD after application of pilocarpine was 2452.71 ± 297.74 *μ*m. The mean pilocarpine-induced lens shift (LS_Pilo_) was 112.29 ± 30.72 *μ*m (*P* < 0.05, Figure 1). Multiple linear regression analysis indicated that LS_Pilo_ was not associated with any of the preoperative parameters listed in Table 1.

Table 2 shows the main postoperative results of all patients at 3 months after surgery. There was a significant improvement in the BCDVA (log MAR value) comparing to the preoperative baseline (*P* < 0.001). For postoperative near vision, UCNVA and DCNVA were 20/40 or better in 62.5% (15/24) and 79.2% (19/24) of eyes, respectively. Comparing to the preoperative baseline, there was no significant change in WTW diameter, CCT, pupil size, and axial length after surgery (all *P* > 0.05). No significant intraoperative or postoperative complications were recorded. Mild posterior capsular fibrosis was noted in 9 eyes (37.50%) at the 3-month follow-up.

**Figure 2:**
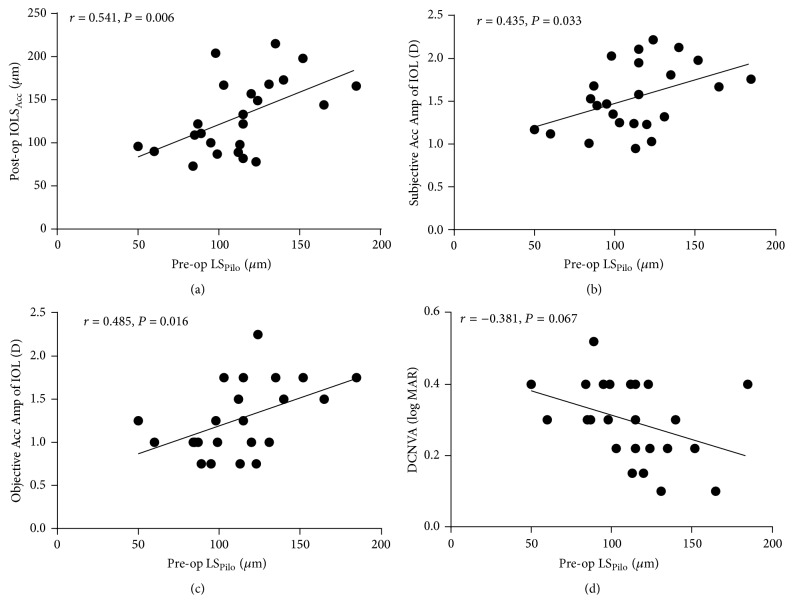
Correlation of preoperative lens shifts induced by pilocarpine (LS_Pilo_) and different postoperative parameters. (a) The preoperative LS_Pilo_ positively correlated with the postoperative intraocular lens shift under an accommodation stimulus (IOLS_Acc_) (*r* = 0.541; *P* = 0.006). (b) The preoperative LS_Pilo_ positively correlated with the postoperative subjective accommodation of Acc-IOLs (*r* = 0.435; *P* = 0.033). (c) The preoperative LS_Pilo_ positively correlated with the postoperative objective accommodation of Acc-IOLs (*r* = 0.485; *P* = 0.016). (d) The preoperative LS_Pilo_ did not correlate with the postoperative distance-corrected near visual acuities (DCNVA) (log MAR value) (*r* = 0.381; *P* = 0.067).

The mean postoperative ACD was 3197.92 ± 349.71 *μ*m at baseline and was 3067.46 ± 341.26 *μ*m at the maximum accommodation stimulus. The mean IOL shift under the accommodation stimulus (IOLS_Acc_) was 130.46 ± 42.71 *μ*m (*P* < 0.05, Figure 1). IOLS_Acc_ was positively correlated with LS_Pilo_ (*r* = 0.541; *P* = 0.006) (Figure 2). The mean postoperative subjective accommodation measured with NPA was 1.54 ± 0.39 D, the mean objective accommodation measured with OQAS was 1.27 ± 0.41 D, and there was a strong correlation between subjective and objective accommodations (*r* = 0.715; *P* < 0.001). There was positive correlation between LS_Pilo_ and subjective and objective accommodation (*r* = 0.412; *P* = 0.022; *r* = 0.466; *P* = 0.045, resp.) (Figure 2). The postoperative DCNVA was correlated with objective accommodation (*r* = 0.488; *P* = 0.016) and IOLS_Acc_ (*r* = 0.427; *P* = 0.025), but not directly with LS_Pilo_ (*r* = 0.381; *P* = 0.067) (Figure 2).

Postoperative objective accommodation and DCNVA were selected as indicators of the efficacy of Acc-IOL implantation. The potential preoperative predictive factors for these two indicators were revealed by multiple linear regression analysis. Objective accommodation was associated with LS_Pilo_ (*β* = 0.380;* P* = 0.016) and AL (*β* = −0.353;* P* = 0.032); the postoperative DCNVA (log MAR value) was associated with pupil diameter (*β* = 0.311; *P* = 0.049) and age (*β* = 0.368; *P* = 0.038).

## 4. Discussion

The great development of cataract surgery has led to an increase in the number of intraocular lenses (IOLs) that attempt to achieve the best uncorrected visual acuity possible at all distances. The multifocal intraocular lenses (MF-IOLs) seem to be dominant in the market in recent years. However, this type of IOL has intrinsic drawbacks of reduced contrast sensitivity, halos around lights, and night glare [1–3], due to its basic principle of redistribution of the light energy with no single focus receiving all the energy as it happens in normal physiological accommodation. The single-optic accommodating intraocular lens (Acc-IOL) is another option for restoration of accommodation in pseudophakic patients after cataract surgery [4]. Current Acc-IOL designs are based on the “focus shift” principle: contraction of the ciliary muscle causes the optic to move anteriorly through various mechanisms, thereby increasing the dioptric power of the eye. However, according to different reports, there was a big variation in the outcomes among individuals after uncomplicated cataract surgery and Acc-IOL implantation [6–10]. The underlying causes remain unknown. Currently there is no effective method to predict the postoperative accommodation based on the preoperative evaluations or established criteria to determine the proper candidate for Acc-IOL implantation.

It is well known that the most accepted model of accommodation is based on the theory of Helmholtz in terms of the interactions between the ciliary muscle, zonule, and lens. There is an increase of curvature and to some extent an anterior movement of in the crystalline lens during accommodation [15]. The prime cause of the development of presbyopia has been interpreted to be lenticular growth and the associated changes in its viscoelastic properties as a function of age [23]. Meanwhile the ciliary muscle maintains most of its contraction power in presbyopic eyes and just declines slightly over age [24]. In senile or age-related cataract patients, the lens loses its properties of deformation due to increased density and rigidity; however, the axial movement driven by ciliary contraction may still exist. In our study, there was a significant translational forward lens shift of about 112 *μ*m under pilocarpine. The amount of pilocarpine-induced lens shift (LS_Pilo_) may indirectly reflect the potential contraction power of ciliary muscles. In addition, we found that LS_Pilo_ was independent of other preoperative parameters including age, which confirms that the contraction power of ciliary muscles may not change as a function of age.

The measurement of the IOL shift under accommodation stimulation has been used to evaluate the accommodation efficiency of Acc-IOLs [8, 18, 25, 26]. Marchini et al. [25] measured the forward shift of the Crystalen® AT-45 Acc-IOL with accommodative effort using ultrasonic biological microscope (UBM) and found that the forward shift could reach 330 *μ*m 6 months after their implantation. Dong et al. [8] observed that the forward mobility of TetraFlex® Acc-IOL under stimulated accommodation with pilocarpine was 337 *μ*m measured with AS-OCT 3 months after surgery. Koeppl et al. [18] indicated that IOL movement may be overestimated when using pilocarpine to stimulate accommodation in aged subjects. Therefore, we measured the IOL shift under physiological accommodation stimulation with AS-OCT. The forward mobility of TetraFlexHD under stimulated accommodation was around 130 *μ*m, which is less than the previous reports listed above. The discrepancy may be attributed to the different designs of Acc-IOLs, accommodative stimulations, as well as the assessment methods adopted. The postoperative IOLS_Acc_ correlated positively with the preoperative LS_Pilo_. The underlying link between these two parameters is that both the cataractous crystalline lens and Acc-IOL are dependent on the ability of ciliary muscle contraction. Therefore, the preoperative LS_Pilo_ might be considered as an indicator of the mobility of the Acc-IOL to be implanted.

In this study, we utilized OQAS to measure objective accommodation to make up for the weakness within the subjective NPA method. The subjective accommodation and objective accommodation were significantly correlated, while the former was slightly higher than the latter. The difference between the subjective and objective measurements of accommodation may be attributed to the inclusion of depth-of-focus effects [27]. Both subjective and objective accommodation correlated positively with the preoperative LS_Pilo_ and postoperative IOLS_Acc_. In addition, the objective accommodation was also associated with the preoperative axial length. This can be explained by the “focus shift” principle of Acc-IOL design. The degree of accommodative effect is related not only to the degree of IOL movement but also to the power of IOL, which largely depends on the axial length [28]. Nawa et al. [29] demonstrated that a 1 mm displacement equates to a 0.8 D change in power in an eye with an axial length of 27 mm, keratometry 7.7 mm, and IOL power 11 D, whereas a 1 mm displacement equates to 2.3 D of change in power in an eye with an axial length of 21 mm, IOL power 30, and the same keratometry. However, in the current study, the Acc-IOL shifts were inadequate to produce the amount of accommodation according to the mathematical calculations. In fact, the accommodation of pseudophakic eye (pseudophakic accommodation) is different from the true accommodation of natural crystal lens in phakia. Sergienko et al. [30] suggested that the accommodative ability of pseudophakic eye was composed of two components: artificial accommodation resulting from axial optic movement and pseudoaccommodation as a consequence of a particular depth of focus, and the proportion between artificial accommodation and pseudoaccommodation of Acc-IOL was nearly 1 : 2. Beside the depth of focus, myopic astigmatism [11], corneal multifocality [31], and aberrations [13] may also contribute to the pseudophakic accommodation. All these factors may account for the difference between the mathematical calculation and clinical measurement of accommodation in pseudophakia.

Most subjects in our study achieved fairly good near vison despite the relatively limited accommodation of Acc-IOLs as measured. This is not surprising to us since patients with monofocal IOL implantation having good uncorrected visual acuity for distance and near vision are occasionally noted from clinical experience. Depth of focus, an intrinsic characteristic of all optical systems that is attributed to aberrations and pupil size, is considered to be one of the most significant contributing factors to near vison in pseudophakic eyes [30]. This was confirmed with the association between DCNVA and pupil diameter in our study. Obviously, all these factors affecting pseudophakic accommodation are responsible for the near vision [11–13]. In addition, decreased near vision ability of pseudophakic patients proportional with age was found by Hayashi et al. [14]. Similar inverse association between the postoperative near vision and age was demonstrated in our study. The aging decay of visual perception is the main causative factor for this phenomenon [14].

This study has some limitations. We did not rule out the possibility of other factors that may affect the postoperative pseudophakic accommodation and near vision, such as dynamic change in pupil size during accommodation, corneal astigmatism, aberrations, and posterior capsular fibrosis. The small sample size and short duration of follow-up also limit the power of this study. Future studies based on a larger population and longer follow-up will be performed to investigate the multiple factors that are associated with the accommodation and visual outcomes of Acc-IOLs.

In conclusion, LS_Pilo_ is an independent preoperative parameter associated with the postoperative Acc-IOL mobility and pseudophakic accommodation. As an indicator of the potential of ciliary muscle contraction, LS_Pilo_ may give valuable information for ophthalmologists in determining the suitable candidates for Acc-IOL implantation.

## Figures and Tables

**Table 1:** Preoperative clinical characteristics in eyes enrolled in this study (*n* = 24).

Factors	
Age, y	63.50 ± 6.86 (48–75)
Sex (% women)	58.33%
Laterality (% right eye)	45.8%
Spherical equivalent (D)	−0.72 ± 1.15 (−2.75 ± 1.88)
BCVA (log⁡MAR)	1.24 ± 0.72 (0.4–2.0)
Axial length (mm)	24.35 ± 2.32 (21.5–27.3)
WTW diameter (mm)	11.56 ± 0.45 (10.7–12.2)
CCT (*µ*m)	513.43 ± 37.48 (450–565)
Mean keratometric value	44.38 ± 1.27 (42.14–46.86)
Pupil size (mm)	4.32 ± 0.55 (3.3–5.4)
Nuclear opalescence	2.98 ± 1.12 (1–5)
Nuclear color	2.92 ± 1.11 (1–5)
IOP (mmHg)	15.14 ± 2.96 (11–20)
ACD (*µ*m)	2565.60 ± 305.50 (2136–3018)
LS_Pilo_ (*µ*m)	112.29 ± 30.72 (50–185)
IOL power (D)	19.92 ± 2.16 (14–25)

D: diopters; BCVA: best-corrected visual acuity; log⁡MAR: logarithm of the minimum angle of resolution; WTW: white-to-white; CCT: central corneal thickness; IOP: intraocular pressure; ACD: anterior chamber depth; LS_Pilo_: lens shift induced by pilocarpine; IOL: intraocular lenses. Results are expressed as means ± standard deviation (range).

**Table 2:** Results of ocular factors at 3 months postoperatively.

Factors	
Spherical equivalent (D)	−0.40 ± 0.74 (−1.02 ± 0.63)
UCDVA (log⁡MAR)	0.26 ± 0.14 (0.1–0.52)
BCDVA (log⁡MAR)	0.22 ± 0.11 (0–0.40)
UCNVA (log⁡MAR)	0.27 ± 0.15 (0.1–0.60)
DCNVA (log⁡MAR)	0.24 ± 0.12 (0.05–0.52)
WTW diameter (mm)	11.60 ± 0.53 (10.8–12.1)
CCT (*µ*m)	521.16 ± 36.55 (458–573)
Pupil size (mm)	4.29 ± 0.59 (3.1–5.5)
Axial length (mm)	24.38 ± 2.29 (21.7–27.2)
Postoperative ACD (*µ*m)	3197.92 ± 349.71 (2785–3961)
Subjective accommodation (D)	1.54 ± 0.39 (0.95–2.22)
Objective accommodation (D)	1.27 ± 0.41 (0.75–2.25)
IOLS_Acc_ (*µ*m)	130.46 ± 42.71 (73–215)
IOP (mmHg)	12.63 ± 2.44 (8.7–18.2)

D: diopter; UCDVA: uncorrected distance visual acuity; log⁡MAR: logarithm of the minimum angle of resolution; BCDVA: best corrected distance visual acuities; UCNVA: uncorrected near visual acuity; DCNVA: distance-corrected near visual acuities; WTW: white-to-white; CCT: central corneal thickness; ACD: anterior chamber depth; IOP: intraocular pressure; IOLS_Acc_: IOL shift under accommodation stimulus. Results are expressed as means ± standard deviation (range).
